# Immunological response to *Brucella abortus* strain 19 vaccination of cattle in a communal area in South Africa

**DOI:** 10.4102/jsava.v89i0.1527

**Published:** 2018-03-29

**Authors:** Gregory J.G. Simpson, Tanguy Marcotty, Elodie Rouille, Abel Chilundo, Jean-Jacques Letteson, Jacques Godfroid

**Affiliations:** 1 Department of Production Animal Studies, University of Pretoria, South Africa; 2 VERDI-R&D, Louveigné, Belgium; 3 National Veterinary College of Toulouse, Toulouse, France; 4 Department of Clinics, Universidade Eduardo Mondlane, Mozambique; 5 Research Unit in Microorganisms Biology, Faculty of Science, University of Namur, Belgium; 6 Department of Artic and Marine Biology, University of Tromsø, Norway

## Abstract

Brucellosis is of worldwide economic and public health importance. Heifer vaccination with live attenuated *Brucella abortus* strain 19 (S19) is the cornerstone of control in low- and middle-income countries. Antibody persistence induced by S19 is directly correlated with the number of colony-forming units (CFU) per dose. There are two vaccination methods: a ‘high’ dose (5–8 × 10^10^ CFU) subcutaneously injected or one or two ‘low’ doses (5 × 10^9^ CFU) through the conjunctival route. This study aimed to evaluate serological reactions to the ‘high’ dose and possible implications of the serological findings on disease control. This study included 58 female cases, vaccinated at Day 0, and 29 male controls. Serum was drawn repeatedly and tested for *Brucella* antibodies using the Rose Bengal Test (RBT) and an indirect enzyme-linked immunosorbent assay (iELISA). The cases showed a rapid antibody response with peak RBT positivity (98%) at 2 weeks and iELISA (95%) at 8 weeks, then decreased in an inverse logistic curve to 14% RBT and 32% iELISA positive at 59 weeks and at 4.5 years 57% (4/7 cases) demonstrated a persistent immune response (RBT, iELISA or Brucellin skin test) to *Brucella* spp. Our study is the first of its kind documenting the persistence of antibodies in an African communal farming setting for over a year to years after ‘high’ dose S19 vaccination, which can be difficult to differentiate from a response to infection with wild-type *B. abortus*. A recommendation could be using a ‘low’ dose or different route of vaccination.

## Introduction

Brucellosis caused by *Brucella abortus* is a widely-distributed zoonosis of importance to public health (Corbel 2006). Animal brucellosis affects mammals, including livestock and wildlife and commonly causes abortion in females and orchitis in males (Chaparro et al. 1990; Dorneles et al. 2015a). In humans, symptoms include fever, malaise, orchitis and a variety of non-specific symptoms (Doganay & Aygen 2003). The KwaZulu-Natal province in South Africa, with a setting similar to the study site, had an estimated prevalence of 0% – 1.5% (Hesterberg et al. 2008). In southern Africa, studies in pastoral production systems have shown the prevalence of brucellosis to be higher with larger herds, extensive movement of animals and co-mingling of herds at common grazing sites (McDermott & Arimi 2002).

In South Africa, where heifer vaccination is mandatory, cattle are seen to be the greatest source of outbreaks (Hesterberg et al. 2008). Detection of disease is done using the Rose Bengal Test (RBT) as the serological screening test and the complement fixation test (CFT) as the confirmatory test. Both tests can give false-positive reactions owing to strain 19 (S19) vaccination (World Organisation for Animal Health 2009). Testing is voluntary, except for dairy cattle, where it is compulsory. Testing frequency depends on resources and animal owners’ motivation. The government has a Bovine Brucellosis Scheme to encourage animal owners to participate in eradicating brucellosis. Vaccination of heifers together with brucellosis testing and slaughter of positive animals is the foundation for control of brucellosis in cattle in endemic areas (Nicoletti 2010). Infected herds are quarantined, infected animals removed and animals are deemed brucellosis-free only after two negative tests at least 3 months apart (*Animal Diseases Act*, Act 35 or 1984). However, in resource-limited settings, slaughter of positive reactors is often not possible because of financial limitations (Moriyón et al. 2004).

Males are not vaccinated because of the potential complication of orchitis (Olsen & Palmer 2014) and the limited role they play in transmission (Olsen & Tatum 2010). Humans who work with these animals or consume their milk and meat are indirectly protected through the vaccination of cattle (Corbel 2006; Godfroid et al. 2011). Two strains are predominantly used for vaccination, where the disease is not properly controlled, as in many low- to middle-income countries (LMICs), S19 is preferred to RB51 (Moriyón et al. 2004).

The World Organisation for Animal Health (OIE) advises vaccinating 5–8 × 10^10^ organisms (‘high’ dose) of S19 to heifers between 3 and 8 months of age (OIE 2016). S19 is effective at inducing an immunological response but, unlike RB51, this response interferes with the serological screening of natural infections (Schuurman 1983). S19 has an O-chain lipopolysaccharide, unlike RB51, that results in antibody persistence (Schurig, Sriranganathan & Corbel 2002). Little is known about the duration of the antibody response to S19 using the government recommended dose of 5 × 10^10^ organisms between 4 and 8 months in heifers and its interference in serological diagnostics in the longer term in a southern African field setting. A reduced dose of 3 ×10^8^ to 5 × 10^9^ organisms (‘low’ dose) can be given by subcutaneous or conjunctival route to decrease the antibody response (Nicoletti 1984; OIE 2016). The administration via the conjunctival route is more difficult, especially in a setting without efficient animal handling facilities. The only similar study in an African setting using a reduced dose of 3 × 10^9^ organisms found that in 92 adult communal cattle the RBT-positive results decreased from 48% at 1 month after vaccination to 2.2% at 9 months and disappeared within 1 year (Schuurman 1983). In Brazil, the same dose was given to adult animals and by 9 months the RBT positives dropped to 0% from 100% at 1 month and indirect enzyme-linked immunosorbent assay (iELISA) to 4% from 100% at 1 month (Poester et al. 2000). In Argentina, a higher but still reduced vaccination dose of 3 × 10^10^ organisms in dairy heifers resulted in 100% buffered plate agglutination test (BPA) positive and 95% iELISA positive at 3 weeks, but 10% BPA positive and 0% iELISA positive at 50 weeks (Aguirre et al. 2002). Other studies have documented antibody and cell-mediated responses to vaccination (Dorneles et al. 2015b; Nielsen & Duncan 1988; Saegerman et al. 1999; Stevens, Olsen & Cheville 1995), but it is difficult to use these studies to make inferences about using serological tests for disease control after vaccination in a communal farming setting as they looked at different indicators of the immune response. These studies also used different colony-forming units (CFUs), vaccination dosages, ages of animals, breeds and settings.

The objective of this study was to follow the serological response to the *Brucella abortus* S19 vaccine (‘high’ dose) in cattle in a rural community bordered by wildlife protected areas using the current government vaccination protocol. We describe the proportion of cattle that seroconverted and the persistence of antibodies in the blood. These results will provide valuable information for understanding brucellosis screening in similar settings in Africa where ‘high’ dose S19 vaccine is used.

## Materials and methods

### Study site

**FIGURE 1:**
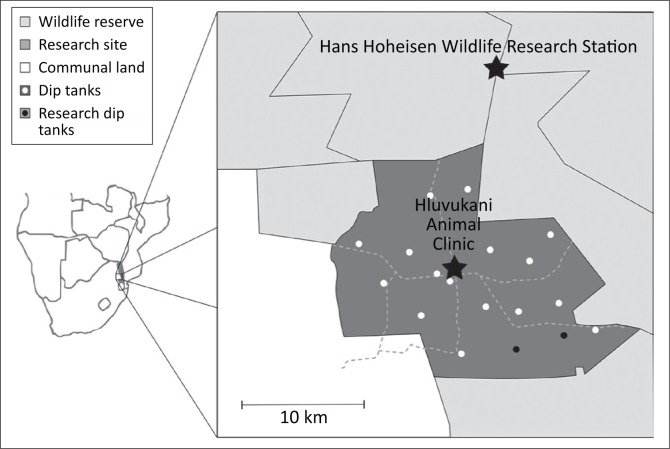
Map of the Mnisi research site. The sampling was done at the two research dip tanks indicated by black dots.

The Mnisi community consists of 30 000 hectares of bushveld savannah on the western border of the Kruger National Park in South Africa and is in the Greater Limpopo Transfrontier Conservation Area (Figure 1). The Hluvukani Animal Clinic, in the middle of the community, was used as the base for the study. A quarter of households in the community, of around 40 000 people, possess cattle (Berrian et al. 2016). As there is close contact between cattle, their products and their handlers, this community is at risk of contracting brucellosis if control measures applied are not adequately preventing the transmission of brucellosis to and between cattle. The area is surrounded on three sides by private and public nature reserves containing wildlife including African buffaloes (*Syncerus caffer*) which are seen as a potential source of brucellosis (Bengis 1998). This study joins other studies in the area that looked at detecting brucellosis in humans, cattle, goats and dogs, investigating the epidemiology of brucellosis in the area. Importantly, we demonstrated that brucellosis was absent in these species in this setting (Simpson, Marcotty, Rouille, Matekwe, Letesson & Godfroid 2017). This unique epidemiological situation allowed us to follow the serological responses induced by the S19 vaccination over time in the absence of natural infection with *B. abortus* infection in a rural communal African setting.

The Mnisi community has an estimated 16 000 cattle in the proximity of 19 dip tanks. Dip tanks were initially constructed to control tick-borne diseases and are used as administrative centres for livestock health surveillance. The study was discussed with the dip tank committees, and six owners volunteered to have their herds enrolled in the study at the Athol and Utha Scheme dip tanks. The cattle enrolled per owner varied from 2 to 35 according to their herd size.

### Study design

This was a longitudinal cohort study following the routine government control programme. Heifers (*n* = 58) with estimated age range between 3 and 12 months (median: 5 months) were vaccinated subcutaneously with 5 mL of *Brucella abortus* S19 vaccine (Onderstepoort Biological Products [OBP], Pretoria, South Africa). This vaccine has approximately 5 × 10^10^ organisms per dose of 5 mL (R. Macdonald [OBP] pers. comm., 15th September 2014). Twenty-nine unvaccinated males of 3–12 months (median: 6 months) in the same herds as the females served as controls. Eight per cent of cases were estimated to be younger than 4 months and 8% were estimated to be older than 4 months.

The immunological response to vaccination was measured by two serological tests: RBT and iELISA. Blood was tested on the day of vaccination (Day 0) and subsequently in 2, 4, 10, 14, 19, 24, 32, 43, 51 and 59 weeks after vaccination. All animals had to be RBT negative at the beginning to be included in the study. Four and half years later, the immunological response was again measured with the aid of these two serological tests and an intradermal Brucellin skin test (ST) in as many cases that could be traced.

### Sampling

Each animal was individually identified. At sampling, 10 mL of blood was collected from the jugular or tail veins. The sera were separated by centrifuging at 1200 g for 10 min within 24 h after blood collection and 1.4 mL of each serum was stored in cryo vials at -20 °C.

### Serological testing

The RBT was performed as described (Alton et al. 1988) and any visible agglutination was interpreted as a positive test result. The sera taken in weeks 2, 4, 10, 14, 43, 51 and 59 after vaccination were also tested with iELISA. The samples taken at 4.5 years after vaccination were tested with both RBT and iELISA. The iELISA used was the IDEXX Brucellosis antibody test kit (IDEXX, Montpellier, France).

### Brucellin skin test

Four and a half years after their vaccination with the S19 vaccine, traced cases had a ST performed. The ST was performed as described (Saegerman et al. 1999) using standardised antigen, prepared from *B. melitensis* B115 rough strain (BRUCELLERGENE OCB^®^, Synbiotics Europe, France). The Brucellin was injected on day 0 and skin thickness was measured on days 0 and 3.

### Statistical analysis

RBT data were analysed in a mixed logistic regression using time and time^2^ as continuous explanatory variables (StatCorp 2009). Individual animals were taken as random effects to account for repeated samplings of the same animals. iELISA data were analysed in a similar model, except for the explanatory variable. Here, a categorical variable was used: 2 months after vaccination (1–3 months) and 1 year after vaccination (10–14 months). The agreement between RBT and iELISA was evaluated using Cohen’s kappa test.

In preliminary models, the effect of age at vaccination (continuous explanatory variable) and that of missing values (multiple imputation) on the serological responses were evaluated and found insignificant. Therefore, they were ignored in the statistical analyses presented in this article.

### Ethical considerations

Ethics approval for the study was obtained from the University of Pretoria, Animal Use and Care Committee (V026-12 and V085-15).

## Results

**FIGURE 2:**
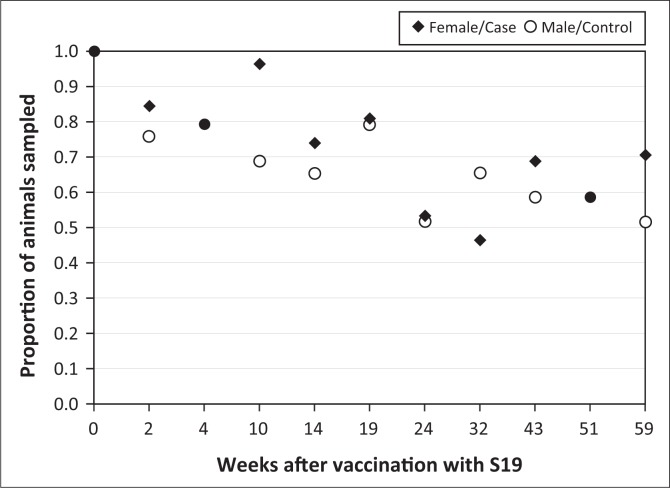
Proportion of animals blood sampled over course of study.

A total of 691 samples were collected from the 58 cases and 29 controls. Sampling success at each sampling date differed through the study and went as low as 53% for cases and 52% for controls at week 22 (Figure 2).

All cases and controls were RBT negative at the beginning of the study on the day of vaccination. One sample of a control animal was RBT positive during the study, but iELISA negative. There were no other control RBT- or iELISA-positive samples. All the control animal study results, when corrected for sensitivity and specificity, were found to be 0% (95% confidence interval [CI]: 0.0–10.4) (Thrusfield 1995).

**FIGURE 3:**
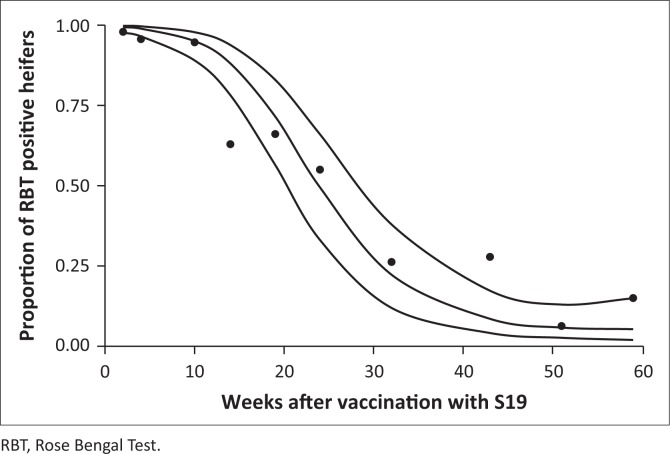
Observed proportion of Rose Bengal Test–positive case results over time using quadratic logistic regression and with 95% confidence intervals.

**FIGURE 4:**
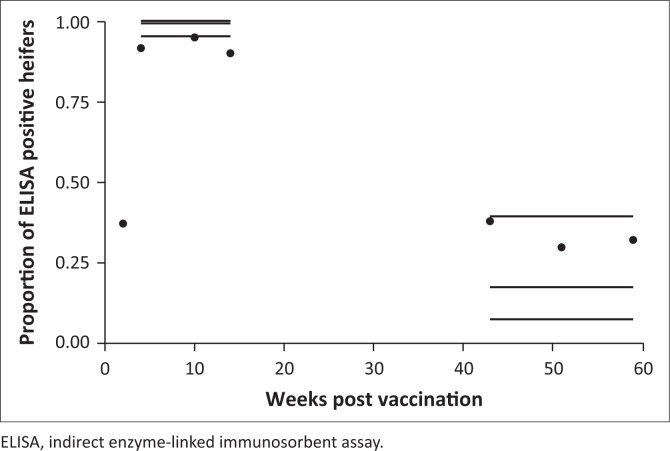
Observed proportion of indirect enzyme-linked immunosorbent assay–positive case results over time using categorical logistic regression and with 95% confidence intervals.

At the second sampling, at 2 weeks, all except one of the 49 cases were sampled and were positive with RBT. All 58 cases tested positive at least once with the RBT within 12 weeks. For three cases, it took 8 weeks to get the first post-vaccination positive result. The percentage of RBT and iELISA positive decreased from their peak after 2 and 8 weeks after vaccination, respectively, but there were still positive animals, 14% for RBT and 32% for iELISA, 59 weeks after vaccination (Figures 3 and 4).

Four and a half years after vaccination, blood samples were taken from all cases that could be traced, which was only 7 (12%). Out of seven cases tested with RBT, iELISA and Brucellin ST, one was positive with RBT and iELISA and three other cows were positive on ST only (Table 1).

**TABLE 1:** Serological and skin test results of seven cases 4.5 years after vaccination.

Animal	RBT	iELISA	ST
1	N	N	N
2	N	N	P
3	N	N	N
4	N	N	P
5	N	N	N
6	P	P	N
7	N	N	P

N, negative; P, positive; ST, skin test; iELISA, indirect enzyme-linked immunosorbent assay; RBT, Rose Bengal Test.

## Discussion

The RBT is seen as the best screening test for brucellosis in this context (Godfroid, Nielsen & Saegerman 2010) and iELISA is seen as a confirmatory test as it is more specific than the RBT (Corbel 2006), both of which are recommended by the OIE. The ST is an assessment of the cell-mediated immunity as opposed to the humoral immunity assessed by the serological tests and is seen as more specific but less sensitive than the RBT and iELISA and more suitable to low prevalence settings (Godfroid et al. 2002; MacDiarmid & Hellstrom 1987; Saegerman et al. 1999).

The expected seroconversion of cases was 100% (Lord et al. 1998). A sample size of 58 cases that all seroconvert is required to obtain 95% as a lower limit of the 95% confidence interval (Thrusfield 1995). The results demonstrated a robust and rapid serological response to vaccination in 100% of animals. Ninety-eight per cent (48/49) of cases seroconverted with RBT by week 2, which is reported in other publications (Stevens, Olsen & Pugh 1995), and all cases seroconverted using iELISA by 12 weeks. Cases took longer to seroconvert to iELISA but all seroconverted at least once to the iELISA within 12 weeks.

The proportion of RBT-positive results decreased rapidly after 8 weeks. This decrease changed after 14 weeks to create an inverse logistic curve that reached a plateau after 43 weeks (Figure 3). This trend is linked to the significant quadratic term of time (time^2^). According to the model, the median seroconversion sample date from RBT positive to negative (proportion of heifers that had turned negative) was 24 weeks after vaccination, whereas 5% of the heifers remained RBT seropositive a year after vaccination. The proportion of iELISA-positive cases dropped from 99% (95% CI: 95% – 99.8%) 4–14 weeks post immunisation to 17% (95% CI: 7% – 39%) 43–59 weeks after vaccination (Figure 4). The response declines more sharply with RBT compared with iELISA (5% RBT-positive and 17% iELISA-positive cases between 10 and 14 months). The RBT is known to mainly detect immunoglobulins M (IgM) and immunoglobulins G (IgG), whereas the iELISA detects solely IgG (Nielsen et al. 2005). Hence, earlier RBT positivity and decline are expected as the IgM response to vaccination is earlier than the IgG response and does not last as long as the IgG (Godfroid et al. 2010). The apparent discrepancy between observed and fitted data in Figure 4 and, to some extent, Figure 3 is because of the random effect (animal) included in the mixed model. There is a substantial agreement between the RBT and iELISA results as shown with a kappa coefficient of 0.63, which indicates that the tests follow a similar pattern over time. The increase in positives from 51 to 59 weeks is because of cases that were not tested in week 51 and were tested in week 59 and found to be positive (1 RBT positive and 4 iELISA positive).

Four and a half years after vaccination, out of seven cases still available for testing (the rest were lost to follow-up owing to movement, slaughter or death from disease), one animal (14%) was positive with RBT and iELISA and three other cows were positive (43%) with the ST only. The fact that one animal was positive on both serological tests and not on the ST, and three were only ST positive, reflects the different immunological responses detected by the different tests. The four positive animals at 4.5 years were vaccinated at 3 (RBT and iELISA positive), 4 (iELISA and ST positive), 5 (ST positive) and 8 (skin test positive) months. Therefore, their positivity was probably not because of being vaccinated after 8 months of age, which has been suggested to be one of the main causes of persistence of an antibody response, although the age estimation is subjective.

This comprehensive response to the vaccine, in a setting similar to other African countries, demonstrates the ability of the vaccine to induce a robust immunological response, which can then be used to assess vaccine coverage assuming an absence of a wild-type pathogen. A concern during the study was that the animals would be subjected to natural infection. Vaccinated heifers that are infected by wild-type *B. abortus* will show a classical ‘boost effect’, which is the hallmark of a secondary immune response (Fensterbank & Plommet 1979). This was not seen in our study. As there was only one RBT-positive sample in the controls and no iELISA-positive controls over the 59-week follow-up, natural infection is unlikely to have interfered with the antibody responses in the cases. The fact that non-vaccinated bulls remained negative also strongly suggests that there was no circulation of wild-type *B. abortus* in this setting throughout the duration of the study. A companion study at the same time by the authors documents that there was indeed no natural infection (Simpson et al. 2017). A ‘boost effect’ seen during disease surveillance should always be thoroughly investigated by veterinary services.

In South Africa, where brucellosis is endemic, vaccination is compulsory but testing is not compulsory; it is however important for owners and veterinary officials to know the status of animals. The fact that 17% of the cases had an iELISA-positive response and 5% had an RBT-positive response about a year after vaccination, and that 4.5 years after vaccination there were still serological and allergenic test positives, shows that there can be immunological persistence to vaccination for many years, which has been previously documented in dairy and beef cattle in Belgium (Saegerman et al. 1999). This is relevant for disease control as these positives may be seen and incorrectly interpreted as natural infections and not vaccine responses as they are detectable so many years after vaccination. If there is confusion over whether animals are reacting from vaccination or natural infection, the infection history of herd, area and animals brought into the herd is of importance. This antibody persistence after vaccination could also cause false-positive reactions with the confirmatory CFT (OIE 2009) that is used in South Africa. Careful interpretation of serological results or the use of multiple tests is necessary (Abernethy et al. 2012). Additional testing includes the use of a competitive ELISA (cELISA), which gives fewer false positives as a result of vaccination (Gall & Nielsen 2004), and ideally culture of *B. abortus*, seen as the gold standard (OIE 2009), in order to confirm the presence of wild-type infection and not the vaccine type. The iELISA uses smooth lipopolysaccharides or O-polysaccharides as antigens, which are also sensitive to the antibodies produced by S19 vaccination, while the cELISA adds a monoclonal antibody that is specific for *Brucella* spp. O-polysaccharides that decrease false positives from vaccination (OIE 2009). While the disease status of individuals is being determined, it would be wise to identify and separate the animals that appear to be infected until their status can be assured or they are slaughtered.

This study used a high vaccine dose of 5 × 10^10^ organisms that is in line with the recommendations of the OIE (5–8 × 10^10^ organisms) (OIE 2016). It revealed a longer and more comprehensive antibody response than a similar study in Zambia that used a reduced dose of S19 (3 × 10^9^ *Brucella* bacteria) and in which no young animals (8–18 months at vaccination) tested RBT positive at 6 months after vaccination (Schuurman 1983). This reduced dose will result in less interference with diagnostics, and has been shown to induce the same level of protection as high doses both experimentally (Fensterbank & Plommet 1979; Plommet Fensterbank & Souriau 1976) and in the field (Nicoletti, Jones & Berman 1978).

Another option is to administer a reduced dose (5 × 10^9^ organisms) via the conjunctival route that yields a lower serological response compared with subcutaneous injection (Nicoletti 1984; OIE 2016; Plommet et al. 1976). This will result in less interference with disease control by serological means but is much more difficult and time-consuming to administer in these settings and requires the animal to be well-restrained to avoid unnecessary trauma to the animal or self-injection.

### Limitations of the study

The study had gaps in the sampling and testing of the animals as it was not always possible to sample an animal, and owing to financial constraints not all samples were tested by iELISA. This gives an incomplete picture of the iELISA results curve.

Ageing of the animals may have been inaccurate because only visual indicators, such as size, condition, teeth present and questioning the owner, were used. Combined with this inaccurate ageing is the variation of genetics, feeding and husbandry amongst the herds in the communal farming setting that results in animals growing at different rates. These variations may result in heifers’ age being underestimated and the heifers being vaccinated outside the 4 to the 8-month window, which in turn may be responsible for a prolonging of the serological and allergenic positive responses, as the older an animal is at vaccination the longer the ST reaction persists (Saegerman et al. 1999).

## Conclusion

This study revealed a comprehensive and rapid antibody response in heifers vaccinated with S19 vaccine in this communal farming setting. Ninety-eight per cent of animals seroconverted to the RBT by week 2 and all cases seroconverted to RBT and iELISA by 12 weeks. The antibody presence decreased in an inverse logistic curve to half of the animals being RBT positive at 6 months and around 5% of animals being RBT positive and 17% being iELISA positive 1 year after vaccination. The serological effects of vaccination therefore can persist for more than a year and possibly several years as seen in one animal when tested 4.5 years after vaccination. This can confuse evaluation of the disease status of a herd by serological testing when there has been a history of vaccination with S19. This must be taken into consideration in making herd disease control recommendations after serological testing for brucellosis.

The constant decrease in serological titres in vaccinated heifers over time, combined with the absence of seroconversion in non-vaccinated bulls and with the absence of clinical signs suggestive of brucellosis (i.e. high rate of abortion), allows us to suggest the absence of brucellosis in the presence of positive serological reactions induced exclusively by S19 vaccination (‘high’ dose). It may be advised to use a ‘low dose’ vaccination that could result in less antibody persistence.
